# How to save Medicare: the anti-aging remedy

**DOI:** 10.18632/aging.100479

**Published:** 2012-08-20

**Authors:** Mikhail V. Blagosklonny

**Affiliations:** Department of Cell Stress Biology, Roswell Park Cancer Institute, Buffalo, NY 14263, USA

**Keywords:** health care, diseases, aging, rapamycin

## Abstract

The unprecedented progress in aging research has revealed that rapamycin, a clinically approved drug, is actually an anti-aging agent, which potentially could be employed to delay age-related diseases, thus extending healthy life span. The possibility of preventing diseases by staying young is remarkable in itself. At the same time this advance could save Medicare as we know it. Here I discuss how anti-aging interventions could solve otherwise intractable political problems without tax increases or curtailment of health care benefits.

## Health care crisis

Social Security and Medicare accounted for 36% of federal spending in 2011, and as baby boomers age, those costs are projected to keep rising. As recently forecasted, the Social Security trust fund will be exhausted in 2033, three years sooner than projected last year. And Medicare's hospital insurance trust fund will be depleted in 2024 [1]. One solution is to cut Medicare and other health care benefits, to narrow treatment options, to slow the growth in benefits somewhat for wealthier recipients. Another solution is to increase taxes (in whatever form) and/or to increase the federal budget deficit. These solutions are political. Here I will discuss a biomedical solution, which can be easily incorporated into their political program by both Democrats and Republicans. Then there will be no dilemma either to increase taxes or to decrease benefits. But first we will discuss what is the cause of the forthcoming crisis.

## Crisis as a side effect of improved health care

As recently noted “Republicans and Democrats are noisily blaming each other for the problems of the popular programs, which provide benefits to more than 55 million people. [1]” Yet, the health care crisis is not the fault of either Republicans or Democrats. The crisis is a “side-effect” of the ever-increasing effectiveness of medicine. That is, the crisis is indirectly due to the marvelous achievements of the modern medicine such as organ transplantation, coronary stents, intensive and emergency care, antibiotics against resistant bacteria, MRI and sophisticated tests, all of which decrease human suffering and allow patients with deadly conditions to live for many years. But this life-saving medicine is also responsible, in part, for increasing health care costs.

**Figure 1:**
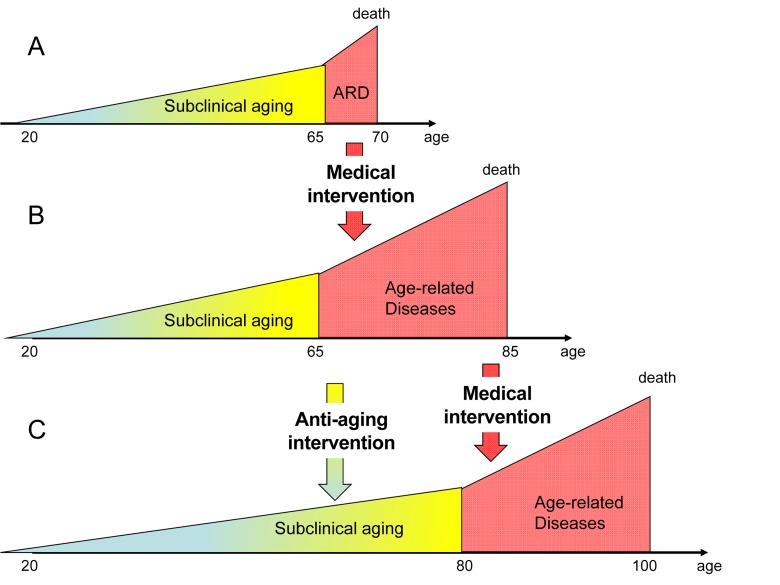
From longer life span to longer health span (and life span) From **A** to **B**: Standard medicine increases lifespan by preventing death from age-related diseases. It simultaneously increases a number of old people suffering from age-related diseases. A ratio healthspan to lifespan is decreased. From **B** to **C**: Anti-aging intervention will slow down aging and delay the onset of age-related diseases. This in theory will restore a ratio of healthspan to lifespan.

First, obviously but not most importantly, these medical options are expensive. For example, organ transplantation may cost hundreds of thousands of dollars. The development of a new antibiotic against drug resistant bacteria requires substantial spending for research. Second, and most importantly, precisely because medicine is becoming so effective in saving lives, this increases a number of elderly patients with chronic and multiple diseases (Figure 1 from A to B), which necessitates multiple treatments all of which cost money. No one dies from aging itself, all humans die from age-related diseases such as cancer, atherosclerosis, hypertension, diabetes, osteoporosis or actually from their complications. So every old person becomes a patient at some point. Medical interventions delay death from age-related diseases, often without curing them. For example, saved by defibrillation from sudden death due to coronary atherosclerosis, a patient can live for many decades (with treatment) and may even die from another age-related disease. With treatment and nursing, patients with macular degeneration, Alzheimer and Parkinson diseases, type II diabetes, hypertension, coronary atherosclerosis, sarcopenia and osteoporosis can live for decades. Cancer is also becoming a chronic disease. Of course this is a great medical and social success. As “a side effect,” however, this increases a number of elderly people with chronic age-related diseases in constant need of health care (who would otherwise have died). Since diseases of aging tend to gradually develop with age, such a patient suffers from several and sometimes many diseases. A combination of obesity, diabetes, atherosclerosis, hypertension, retinopathy, osteoporosis is very common. So there is simultaneously an increase of the number of diseases afflicting each elderly person and an increase in the number of such patients.

In summary, current medicine is effective in preventingdeath from age-related diseases without delaying their onset, thus increasing the number of people with age-related diseases and the number of diseases afflicting each elderly person. In addition, each disease of aging is now treated separately, which is costly and can lead to unavaoidable adverse effects. For example, chemotherapy, used for cancer treatment, has a negative impact on normal tissues and organs. And vice versa, insulin, which is used for treatment of diabetes, is a pro-aging factor [2] and may accelerate some pathologies such as cancer [3], [4]. (Note: In contrast, due to some anti-aging activities, the anti-diabetic drug metformin prevents cancer [5]). One solution is to delay age-related diseases, thus extending healthy life span. But is it possible?

## Slow aging is manifested as healthy aging

There is a misconception that an anti-aging medicine would increase the number of chronically ill people because they are old. On the contrary, it would decrease the ratio of unhealthy to healthy population (Figure 1C) because an anti-aging medicine will delay the onset of aging, diseases and their complications at older age [6-8]. Fast-aging animals (mice) develop diseases of aging fast, whereas slowly aging organisms such as humans acquire these diseases at 40 times older age than mice. Centenarians, people who live more than 100 years, age slowly and generally experience good health until very old ages, when diseases that kill them finally develop [8-12]. Furthermore, the period of morbidity is not only delayed but also shortened [8]. (Perhaps, extremely old (chronologically) patients are nor treated vigorously compared with younger patients).

In the past, most people died (from incidents, infections, malnutrition and homicide) before they achieved the age of age-related diseases. For example, in the 17th century in London, only 25% of people survived until the age of 26 (see for references [7]). By the mid of 20th century civilization and medicine allowed most people to live long enough to die from aging or strictly speaking from age-related diseases (Figure 1A). Still until very recently people died soon after they reached the age of age-related diseases from the complications of these age-related diseases (Figure 1 A). Now effective medical interventions can keep a patient alive despite age-related diseases (Figure 1B). This increases the ratio of unhealthy to healthy population (Figure 1B). What is needed is to delay age-related diseases (Figure 1C). And by lucky co-incidence, this could be done right now, potentially preventing the health care crisis (see text).

Slowing down aging both increases lifespan and postpones diseases. One may even say that **anti-aging interventions increase lifespan by postponing diseases**. Thus, calorie restriction slows aging and delays the development of all age-related diseases in mammals including non-human and human primates [13-18]. And vice versa excessive nutrition that causes obesity and accelerates aging also accelerates development of all age-related diseases from type II diabetes to atherosclerosis to cancer. A mere reduction of visceral fat decreases mortality [19]. Yet, severe calorie restriction may cause malnutrition. It may be possible to use a calorie-restriction mimetic such as rapamycin instead of calorie restriction. There is evidence that rapamycin can slow aging and delay onset of age-related diseases.

## Aging and Target of Rapamycin (TOR)

It was long thought that aging is caused by accumulation of random molecular damage and wear and tear. Accordingly, it was assumed that diseases can be treated but aging cannot. A rapidly increasing number of studies has convincingly established that inhibition of certain signal-transduction molecules extends life span in diverse species [20-26]. These proteins form signaling pathways, which sense nutrients (glucose, fatty acids, amino acids), insulin and other hormones, oxygen, cytokines and growth factors. Activation of such nutrient-sensing pathways promotes growth and, when growth is completed, aging [27]. The nutrient-sensing and growth-promoting TOR (Target of Rapamycin) stands out for four important reasons. First, most of pro-aging and anti-aging molecules can be diagrammed as part of the TOR pathway [28, 29]. Second, mammalian TOR (mTOR) links cellular and organismal aging [28, 30]. Thus, mTOR is involved in cellular aging [31-33]. Inhibition of mTOR suppresses conversion of post-mitotic cells into senescent cells [34, 35]. In resting cells, re-activation of mTOR causes senescence (geroconversion) [36]. Rapamycin prevents hyperactivation and exhaustion of stem cells in the organism [37, 38]. Third, numerous preclinical studies revealed that mTOR is involved in most age-related diseases including including cancer, atherosclerosis, neurodegeneration and age-related macular degeneration [28, 29, 39-43]. Fourth, and most importantly, rapamycin and its analogs (rapalogs) are clinically approved drugs.

## Rapamycin and other rapalogs

For a decade, rapamycin (Sirolimus) and its analogs have been used in high doses in transplant patients. At high and chronic doses, in combinations with immunosuppressants (in order to prevent transplant organ rejection), rapamycin has some reversible side effects. As a “side effect” rapamycin prevents cancer in renal transplant patients [44-46]. There are some “therapeutic side effects” such as lipolysis [40]. There is a misconception that rapamycin may increase risk of cancer and lymphomas. Instead, rapamycin and other rapalogs prevent and treat cancer and lymphomas. Rapamycin prevents many age-related diseases in animal models. In patients, oral rapamycin decreases atherosclerotic re-stenosis [47-49]. Finally, it slows aging and extends life span in flies [50, 51] and mice [52-57].

As an anti-aging drug, however, rapamycin should be used at low doses and intermittent schedules [58, 59] (“Intermittent rapamycin” in preparation). In fact, intermittent therapy with rapamycin still extends lifespan in mice [54, 55]. Similarly, intermittent calorie restriction prolongs life span in rodents. Low doses, intermittent administration and rational combinations with such drugs as metformin (in contrast to immunossuprressants) would distinguish anti-aging schedules of rapamyicin from its use to prevent organ rejection. Doses and schedules always make the difference. Consider arsenic, the most famous poison, used for millennia by murderers. In different doses and schedules, arsenic is now used as the most effective treatment for acute promyelocytic leukemia. Potassium chloride is one of the most useful drugs widely used in medicine. In different administration, potassium chloride is also used in lethal injections for capital punishment. In comparison with other drugs, rapamycin is exceptionally non-toxic, and it cannot be possibly used in lethal injections. The oral dose that is lethal to mice cannot be actually achieved since LD50> 2500 mg/kg. (And this is thousand times more than even a high therapeutic dose). A single dose of rapamycin is not lethal at any dose and furthermore has no side effects in healthy volunteers, providing one of rationales for intermittent schedules. Of course, additional clinical trials of low doses of rapamycin will be necessary to demonstrate that it decreases incidence of age-related diseases. I need to emphasize that there is no evidence yet that rapamycin increases human lifespan. (This would take a human life span to demonstrate). But such evidence is not needed. For practical purposes, to safe Medicare, it is important to delay age-related diseases. As discussed in detail [40], such a clinical trial would take just a couple of years to conduct. And if all diseases will be delayed, then both health span and life span will be increased.

## How to implement and future developments?

Government and public efforts have been very effective in reducing smoking in the U.S. This was achieved despite the fact that smoking is highly addictive! In comparison, wide introduction of low/intermittent doses of the anti-aging drug rapamycin, which by the way decreases incidence of smoke-related cancer in mice on 90% (!)[60], seems less challenging. In general, this would be comparable to the introduction of vaccination, which government and other programs have very effectively carried out. And introduction of rapamycin is just a first step in the development of anti-aging interventions. A program of how to extend life span in our lifetime was recently discussed [61].

## Closing remarks: this seems to be the most civilized solution

Everyone agrees that the ever-rising costs of Medicare must be slowed. Of course the costs can be reduced in part by making the health care system more effective, eliminating any kind of abuse of the health care system by insurance companies, the industry and care providers. These issues are well addressed by politicians. Yet, the costs can be slowed but cannot be frozen. Unfortunately, the only way to stop the rising costs of Medicare completely is to prevent the use of more effective (and expensive) medical options and to stop further biomedical research. This draconian option would accelerate the mortality of the sickest elderly, further decreasing Medicare costs. Of course this is unacceptable. So costs must continue to rise. But this wouldn't necessarily lead to a fiscal crisis, given that anti-aging medicine could increase health span and therefore the ratio of healthy to unhealthy individuals in the elderly population. In conjunction with the increase in health span, the age of retirement could be increased. This would increase federal revenues and provide a means to cover increasing costs of Medicare. In any case, the eligibility age for full benefits is now gradually increasing. For those born after 1960, it will be 67. Some politicians would increase it further, allowing it to rise along with increases in longevity. Yet, although longevity is increasing, the rapid rise in Medicare costs is due to prevention of death from age-related diseases, not to prevention of diseases themselves. Anti-aging interventions may postpone diseases, thus naturally increasing the age of retirement, because at 87 a person would be biologically 67 and feel as healthy and energetic as he or she currently does at 67.

## Summary

1. Anti-aging interventions may increase health span, increase the age of disability allowing chronologically older people to be biologically younger. This naturally increases the age of retirement, increasing revenue without increasing taxes.
2. Currently, there is no other sensible solution. The alternatives, both untenable, are either to let elderly (unhealthy) people die by drastically limiting medical benefits, or perennially to increase taxes.
